# Lipid Droplets Metabolism Mediated by ANXA7‐PPARγ Signaling Axis Regulates Spinal Cord Injury Repair in Mice

**DOI:** 10.1002/advs.202417326

**Published:** 2025-02-25

**Authors:** Lu Chen, Haoran Liu, Linlin Jiang, Zihang Wang, Yong Chang, Na Li, Shiqing Feng

**Affiliations:** ^1^ Orthopaedic Research Center of Shandong University Department of orthopaedics Qilu Hospital of Shandong University #107 Wenhua West Road 250012 Jinan Shandong China; ^2^ School of Basic Medical Sciences Shandong University #44 Wenhua West Road 250012 Jinan Shandong China; ^3^ The Second Hospital of Shandong University #247 Beiyuan Street Jinan Shandong 250000 China

**Keywords:** ANXA7, lipid droplet, oxidative stress, PPARγ, spinal cord injury

## Abstract

Spinal cord injury is characterized by high incidence and high disability, and the specific targets and drugs have not yet been explored. Lipid droplet is a type of organelles that regulates lipid metabolism and oxidative stress. And the regulatory mechanisms of lipid droplets on spinal cord injury remain unclear. Herein, it is found that GTPase activation of Annexin A7 (ANXA7) promotes the up‐regulation of genes related to lipid droplet formation. ANXA7 can interact with peroxisome proliferator‐activated receptor gamma (PPARγ) to enhance the stability of PPARγ, and promote lipid droplet formation and interaction with mitochondria through promoting Perilipin 5 expression. Then, oxidative stress and lipid peroxidation are inhibited due to the promotion of nuclear factor erythroid 2‐related factor 2 (NRF2) nuclear translocation and expression of glutathione peroxidase 4 (GPX4). ANXA7 activation promotes lipid droplet formation and mitochondria‐lipid droplet interaction by enhancing nuclear translocation of PPARγ, which contributes to inhibiting lipid peroxidation and neuron damage. Furthermore, activation of PPARγ can promote neural function recovery and spinal cord repair in mice. The focus of this study is to investigate the effects of lipid droplets regulated by ANXA7/PPARγ, providing new targets and strategies for spinal cord injury.

## Graphical abstract

This work proposes a new mechanism for the regulation of LDs metabolism by ANXA7/PPARγ. ANXA7 can interact with PPARγ to promote its stability. ANXA7 activation promotes PPARγ nuclear translocation, thus promoting LDs formation and interaction with mitochondria by promoting PLIN5 expression. Moreover, ANXA7/PPARγ can also inhibit oxidative stress and lipid peroxidation by promoting nuclear translocation of NRF2 and GPX4 expression.

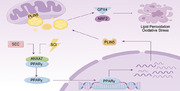

## 1 Introduction

Spinal cord injury (SCI) is a traumatic neurological disease with high disability mortality, which brings a heavy burden to the family and even the society.^[^ ^1^ ^]^ Previous studies have reported that only <1% of patients suffering from SCI could get full neurofunction recovery.^[^ ^2^ ^]^ The lack of understanding about the molecular pathological mechanism of spinal cord injury and precise machinery dysfunctional of neurons has substantially increased interest in damage repair of neurons in recent years. That the molecular pathological mechanism of spinal cord remains unclear and the response to injury is complex, leading to the current non‐specific treatment methods.^[^ ^3^, ^4^, ^5^ ^]^ General surgical treatment could only restore the anatomical integrity and stability of the spine, but not on the recovery of nerve function. At present, the commonly used drugs could only inhibit the acute stress response and have limited effect on nerve repair. And the specifically targeted drugs for the SCI treatment are still blank.^[^ ^6^ ^]^ Therefore, it is important for the SCI research field to elucidate the molecular pathological mechanism of neuron damage and explore specific drug targets.

Lipid Peroxidation (LPO) caused by increased reactive oxygen species (ROS) at the injured part is an important factor in the SCI repair difficulties and plays a key role in the occurrence and development of secondary injury.^[^ ^7^, ^8^ ^]^ However, the specific LPO regulatory molecular mechanism after spinal cord injury is unclear. Lipid droplet (LD) is the nexus to keep the intracellular lipid metabolism balance.^[^ ^9^ ^]^ Under normal physiological conditions, LDs are involved in cellular lipid metabolism and membrane transport, and play a prominent role in alleviating lipid toxicity, anti‐inflammatory and antioxidant stress, etc.^[^ ^10^, ^11^ ^]^ However, LDs have always played a “negative role” in neurological diseases. For example, LDs accumulation in the brains of patients with Alzheimer's disease inhibits the homeostasis and regeneration of neural stem cells.^[^ ^12^ ^]^ LDs have been recognized as an indicator of peripheral inflammation. And LD accumulation in glia has been found under the conditions of brain aging and disease.^[^ ^13^ ^]^ Previous studies have shown that primary neurons usually could not produce LDs under normal physiological conditions, and lipid toxicity after nerve injury is mainly buffered by Schwann cells and microglia.^[^ ^14^, ^15^ ^]^ However, the regulation and mechanism of LDs in primary neurons during spinal cord injury have not been studied.

Annexin A7 (ANXA7) is a member of the annexin family. It binds with calcium ions and phospholipids to exert calcium‐activated GTPase activity, which can regulate a variety of physiological processes in cells.^[^ ^16^ ^]^ ANXA7 could regulate organelle interaction and the balance of lipid metabolism, which plays an important role in the regulation of the injury in central nervous system.^[^ ^17^ ^]^ Our previous studies demonstrated that ANXA7 is primarily expressed in neurons rather than glial cells in central nerve system.^[^ ^18^ ^]^ The expression level of ANXA7 decreases significantly after spinal cord injury, but ANXA7 overexpression or activation of its GTPase activity could inhibit neuronal apoptosis effectively. However, detailed regulatory mechanisms of ANXA7 on SCI need to be further explored.

Nuclear receptor peroxisome proliferator‐activated receptor gamma (PPARγ) is a member of the peroxisome proliferator‐activated receptor family. It could regulate lipid metabolism and oxidative stress.^[^ ^19^, ^20^ ^]^ Studies have shown that PPARγ does not only regulate lipid metabolism,^[^ ^21^, ^22^, ^23^ ^]^ but also inhibits inflammation and oxidative stress in the central nervous system. However, the regulatory effects and mechanisms of PPARγ on spinal cord injury remain to be clarified. In this study, we proposed a new regulatory mechanism of how ANXA7 regulate LDs metabolism to promote spinal cord injury repair via interacting with PPARγ.

## 2 Results

### 2.1 Activation of ANXA7 Promotes LDs Formation and Inhibit Lipid Peroxidation

**Figure 1:**
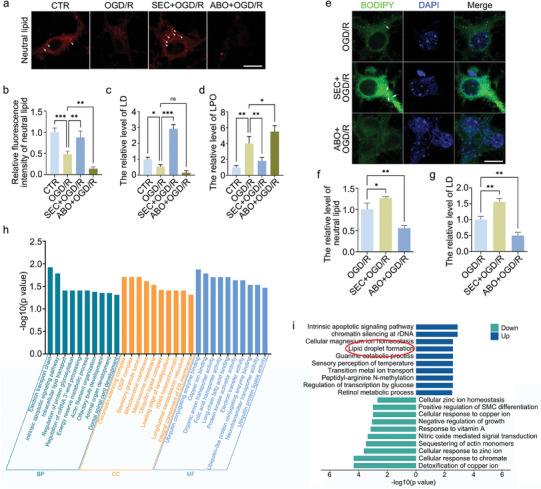
Lipid droplets exist in neurons and activation of ANXA7 promotes lipid droplets formation. a–c) The OGD/R model was constructed on primary neurons after SEC or ABO treatment for 1 h. The neutral lipid dye HCS LipidTOX was used to detect the levels of neutral lipids and lipid droplets in different groups. Scale bar: 10 µm. d) The OGD/R model was constructed on neurons treated with SEC or ABO for 1 h, and the lipid peroxidation (LPO) kit was used to detect the lipid peroxidation level of neurons. e–g) Lipid droplets in neurons were stained by BODIPY 493/503 after OGD/R treatment with SEC or ABO. Scale bar: 10 µm. h) GO analysis was performed between SEC+OGD/R group and OGD/R treatment group after 4D proteomic sequencing. i) The results of up/down regulated activities in biological process (BP) analysis. The data were presented as mean ± SD, and ANOVA was used for analysis. ^*^ *p* < 0.05, ^**^ *p* < 0.01, ^***^ *p* < 0.001, n = 3.

Previous studies have shown that ANXA7 has a regulatory effect on lipid metabolism. We examined the regulatory effect of ANXA7 on LDs in neurons and found that ANXA7 activation by SEC could promote LDs formation and rescue lipid peroxidation caused by oxygen–glucose deprivation/reoxygenation (OGD/R) treatment, while ABO, the inhibitor of ANXA7, had the opposite effect (**Figure** **1**a–d). Meanwhile, BODIPY staining used for indicating neutral lipid in neurons showed similar results (Figure 1e–g). To further investigate the regulatory mechanism of ANXA7 on spinal cord injury, OGD/R was performed on mouse primary neurons treated with activator SEC, and 4D proteomic sequencing was conducted. The results of GO analysis showed that the process of intracellular lipid transport was enriched significantly, and LDs formation was up‐regulated after SEC treatment on neurons (Figure 1h,i).

### 2.2 ANXA7 Activation Promotes the Expression of NRF2 and GPX4, and Inhibits Oxidative Stress and Lipid Peroxidation

**Figure 2:**
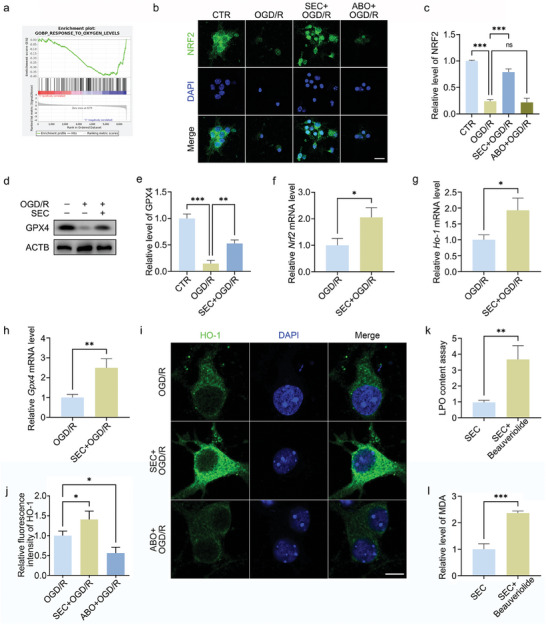
ANXA7 activation could suppress oxidative stress induced by OGD/R treatment. a) GSEA analysis after 4D proteome sequencing between SEC+OGD/R and OGD/R treatment group. b,c) OGD/R model was constructed on neurons after SEC or ABO treatment for 1 h, and NRF2 protein level was detected by immunofluorescence. Scale bar: 20 µm. d,e) The OGD/R model was constructed on neurons after SEC treatment for 1 h, and the GPX4 protein level was detected by WB. f–h) OGD/R model was constructed on neurons after SEC treatment for 1 h, and mRNA levels of *Nrf2*, *Ho‐1*, and *Gpx4* in different treatment groups were detected by qPCR. i,j) HO‐1 fluorescent staining on neurons after OGD/R with/without SEC or ABO treatment. Scale bar: 10 µm. k,l) LPO and malondialdehyde (MDA) levels were detected in neurons treated with SEC or combination of SEC and Beauveriolide III for 1 h after OGD/R. The data were presented as mean ± SD, and Student's T‐test was used for analysis between two groups, while ANOVA was used among more than two groups. ^*^ *p* < 0.05, ^**^ *p* < 0.01, ^***^ *p* < 0.001, n = 3.

Nuclear factor erythroid 2‐related factor 2 (NRF2), also called NFE2L2, is an intracellular REDOX sensor. The expression level of NRF2 is maintained low under normal physiological conditions. However, NRF2 will translocate to the nucleus when cells are attacked by ROS to mobilize the expression of anti‐oxidative stress‐related genes, such as heme oxygenase‐1 (HO‐1), and inhibit oxidative stress. 4D protein sequencing and GSEA analysis showed that ANXA7 activation was positively correlated with response to oxygen levels (**Figure** **2**a). Immunofluorescence experiments confirmed that SEC, the activator of ANXA7, promoted NRF2 nuclear translocation, while the inhibitor ABO had the opposite effect (Figure 2b,c). Glutathione peroxidase 4 (GPX4) is an intracellular selenoprotein antioxidant enzyme which is an important regulator of lipid peroxidation. It has the ability to clear membrane lipid hydroperoxide products and prevent oxidative stress. Studies have shown that a variety of molecules could inhibit lipid peroxidation by restoring the activity of GPX4, and regulate the ferroptosis of neurons after spinal cord injury. In this study, we found that activation of ANXA7 could promote GPX4 expression and rescue GPX4 downregulation caused by OGD/R treatment (Figure 2d,e). Furthermore, SEC treatment upregulates the mRNA levels of *Nrf2*, *Ho‐1*, and *Gpx4* (Figure 2f–h), and increases HO‐1 protein level according to immunofluorescence (Figure 2i,j). Above results suggest that activation of ANXA7 could inhibit oxidative stress and lipid peroxidation by regulating NRF2 pathway and GPX4. We also used lentivirus to regulate the expression of ANXA7 and detected its effect on anti‐oxidative stress‐related proteins. And, similarly, expressions of PPARγ, PLIN5, and NRF2 were changed along with ANXA7 upregulation or downregulation (Figure S1a–j, Supporting Information). However, when we use a LDs formation inhibitor Beauveriolide III, the anti‐lipid peroxidation effect of SEC was inhibited (Figure 2k,l). These results indicate that the inhibitory effect of ANXA7 on lipid peroxidation depends on LDs formation.

### 2.3 ANXA7 Interacts with PPARγ and its Activation Could Increase the Expression of PPARγ

**Figure 3:**
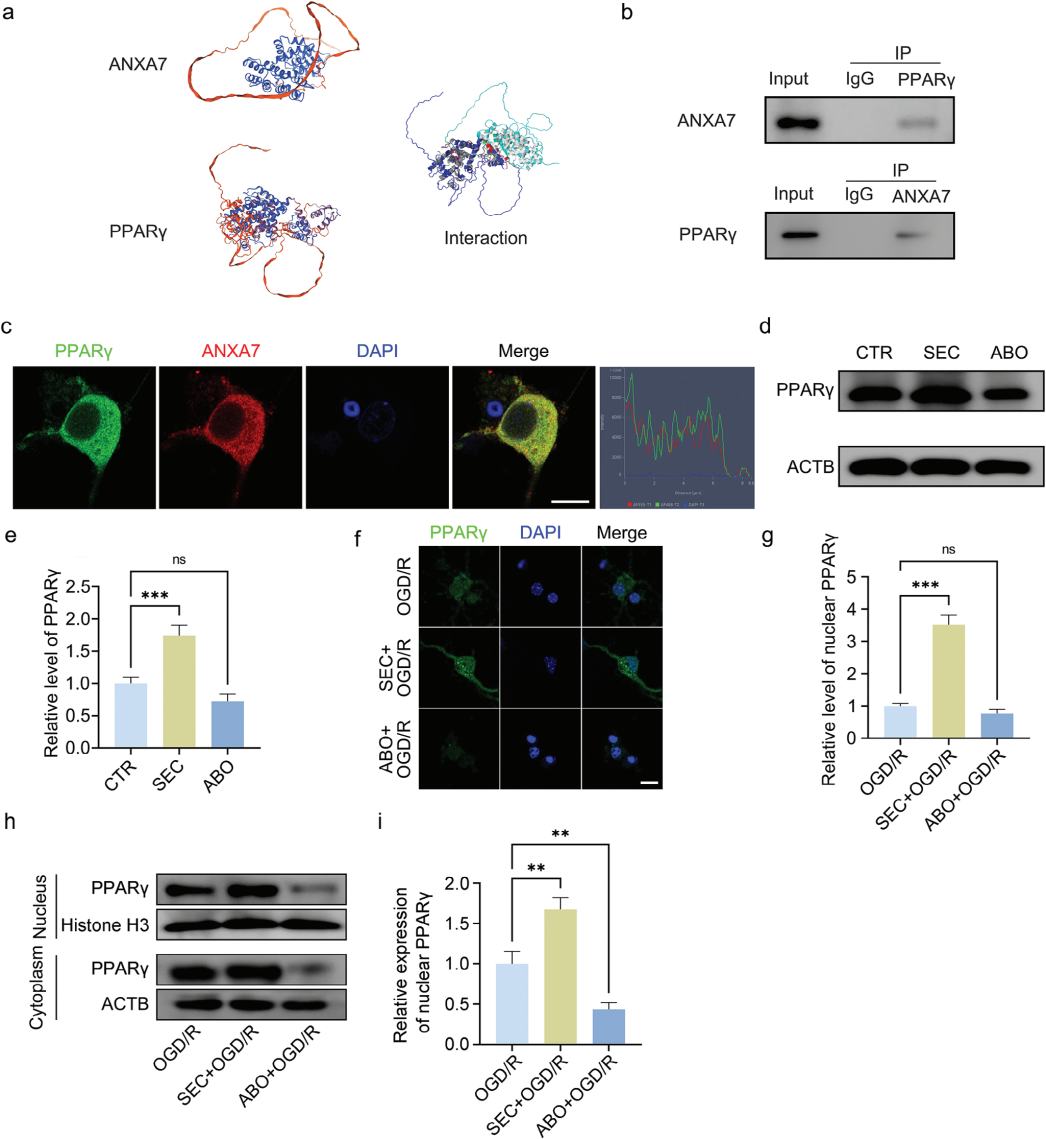
ANXA7 could interact with PPARγ. a) SWISS‐MODEL/Vasker Lab/PDBePISA websites were used to predict structure of ANXA7, PPARγ, and 3D structure diagram of their interaction. b) Co‐immunoprecipitation was used to detect the interaction between ANXA7 and PPARγ. c) Immunofluorescence was used to detect the colocalization of ANXA7 and PPARγ, and the colocalization analysis was conducted. Scale bar: 10 µm. d,e) The protein level of PPARγ was detected by WB after treatment SEC or ABO for 1 h. f,g) Neurons were treated with SEC or ABO for 1 h before OGD/R, and immunofluorescence was used to detect the nuclear level of PPARγ. Scale bar: 10 µm. h,i) Nuclear expression of PPARγ was detected by WB. The data were presented as mean ± SD, and ANOVA was used for analysis. ^**^ *p* < 0.01, ^***^ *p* < 0.001, n = 3.

Previous studies reported that the transcription factor PPARγ could regulate fatty acid metabolism, and affect LDs production as well as oxidative stress.^[^ ^24^, ^25^ ^]^ In order to explore the interaction between ANXA7 and PPARγ, bioinformatic analysis was carried out to predict the interaction model (**Figure** **3**a). The results showed that ANXA7 could interact with PPARγ with many interaction sites, which was also demonstrated by co‐immunoprecipitation and immunofluorescence (Figure 3b,c). Moreover, ANXA7 activation promoted PPARγ expression and its nuclear translocation while ANXA7 inhibition suppressed its expression (Figure 3d–g), which were also proved by the results of WB (Figure 3h,i). The similar results could also be observed in neurons with ANXA7 upregulation (Figure S3a,b, Supporting Information). Thus, we speculate that ANXA7 is likely to regulate LDs formation by interacting with PPARγ, but the function of PPARγ needs to be further studied and the mechanism remains to be clarified.

### 2.4 PPARγ was Downregulated After Spinal Cord Injury, and Activation of PPARγ Promoted Neuronal Survival via Inhibiting Lipid Peroxidation and Oxidative Stress

**Figure 4:**
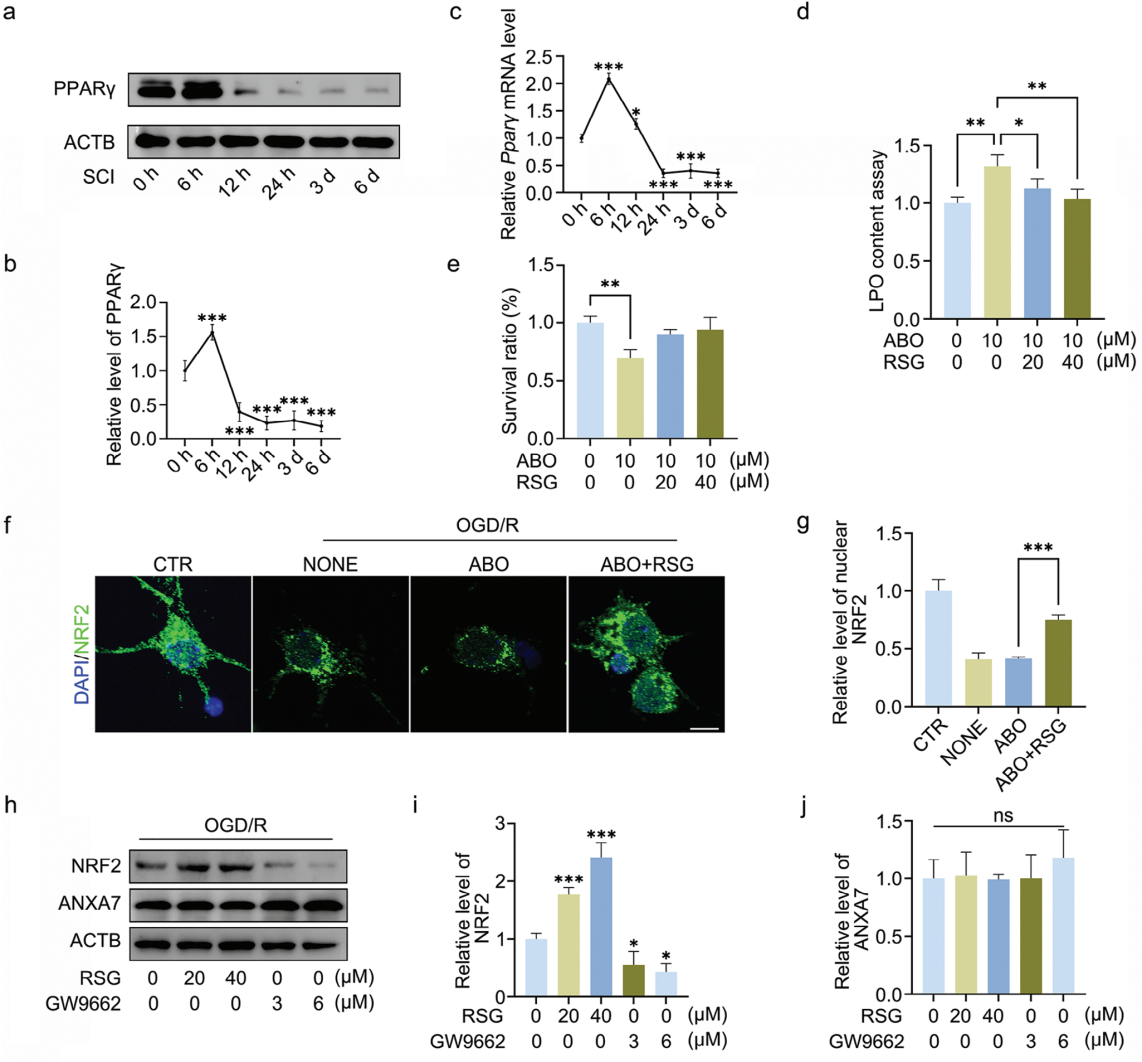
Expression changes of PPARγ after spinal cord injury and its regulatory effect on NRF2. a,b) The mouse model of spinal cord injury was constructed. The spinal cord tissue was extracted and the protein level of PPARγ was detected by WB on different time point after spinal cord injury. c) The mouse model of spinal cord injury was constructed, and qPCR was used to detect the mRNA level changes of *Pparγ* on different time point after spinal cord injury. d) Neurons were treated with rosiglitazone and ABO separately or in combination for 1 h, and LPO levels of the neurons were detected after OGD/R treatment. e) Neurons were treated with rosiglitazone and ABO separately or in combination for 1 h, and the survival ratio of neurons was detected by CCK8 after OGD/R. f,g) Rosiglitazone and ABO were used to treat neurons separately or in combination for 1 h, and immunofluorescence was conducted to detect nuclear translocation level of NRF2 after OGD/R. Scale bar: 10 µm. h–j) Neurons were treated with different concentrations of rosiglitazone and GW9662 for 1 h, respectively. After the OGD/R model was constructed, the protein levels of ANXA7 and NRF2 were detected by WB. The data were presented as mean ± SD, and ANOVA was used for analysis. ^*^ *p* < 0.05, ^**^ *p* < 0.01, ^***^ *p* < 0.001, n = 3.

To reflect the changes of PPARγ expression more accurately, the mouse spinal cord injury model was constructed, and the expression of PPARγ on protein and mRNA levels were examined at different time points after spinal cord injury. The results showed that PPARγ expression was increased in a short period (within 6 h) after spinal cord injury and significantly decreased later, with the lowest expression being at 24 h after spinal cord injury (**Figure** **4**a–c). In addition, we found that rosiglitazone (RSG), the activator of PPARγ, was able to rescue ABO‐induced elevated lipid peroxidation levels and increased neuronal survival ratio (Figure 4d,e). As previously shown in Figure 2b, the activity of ANXA7 could influence the expression of NRF2, and ANXA7 overexpression also has a similar effect (Figure S2c,d, Supporting Information). Moreover, rosiglitazone could also rescue the inhibition of NRF2 nuclear translocation caused by ABO (Figure 4f,g). We also found that PPARγ activation increased NRF2 levels, while its inhibition suppressed NRF2 expression, but changes in PPARγ activity did not alter ANXA7 expression levels (Figure 4h–j).

### 2.5 ANXA7 Promotes LDs Formation and Inhibits Lipid Peroxidation by Increasing PPARγ Stability

**Figure 5:**
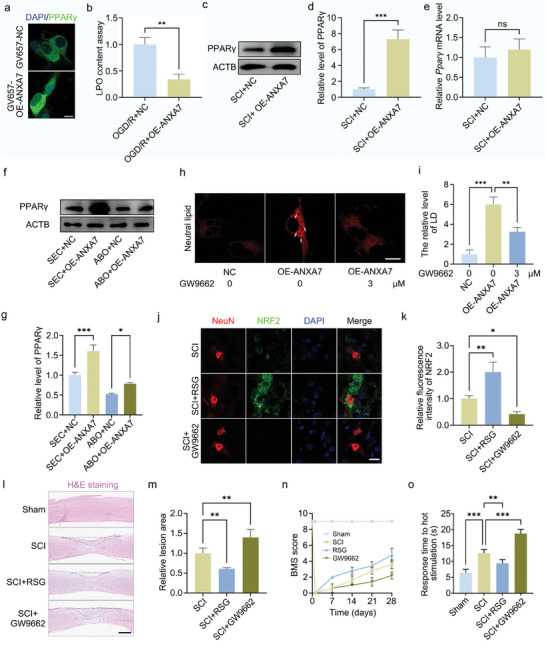
Regulation of ANXA7 to PPARγ and the effect of PPARγ on spinal cord injury repair. a,b) OGD/R model of neurons was constructed after transfected with GV657‐negative control or GV657‐OE‐ANXA7 plasmids. Then the protein expression of PPARγ and LPO content were detected by immunofluorescence and LPO assay kit. Scale bar: 10 µm. c–e) The spinal cord injury mouse model was established. After 4 days, the spinal cord tissue was extracted and PPARγ was detected by WB and qPCR in different treatment groups. f,g) After transfected with ANXA7 overexpression lentivirus, neurons were treated with/without SEC or ABO for 1 h before OGD/R treatment, and protein level of PPARγ was detected by WB. h,i) After transfected with ANXA7 overexpression lentivirus, neurons were treated with/without GW9662 for 1 h before OGD/R treatment, and lipid droplet levels were detected. Scale bar: 10 µm. j,k) Mice were pretreated with rosiglitazone or GW9662, and the expression of NRF2 were detected by immunofluorescence 4 days after SCI. Scale bar: 10 µm. l,m) Mice were pretreated with rosiglitazone or GW9662, HE staining was conducted on spinal cord sections and the lesion area was measured. Scale bar: 500 µm. n) BMS score analysis on mice treated with rosiglitazone or GW9662 after spinal cord injury for 0, 1, 7, 14, 21, 28 days. o) Response times to hot stimulation in mice treated with rosiglitazone or GW9662 after spinal cord injury for 28 days. The data were presented as mean ± SD, and Student's T‐test was used for analysis between two groups, while ANOVA was used among more than two groups. ^*^ *p* < 0.05, ^**^ *p* < 0.01, ^***^ *p* < 0.001, n = 3.

Transfection of GV657‐ANXA7(ANXA7 over‐expression) plasmid could increase the protein level of PPARγ and inhibit lipid peroxidation (**Figure** **5**a,b). And the ANXA7 overexpression lentivirus was also used to evaluate the regulatory effect of ANXA7 on spinal cord injury in mice. We found that ANXA7 overexpression in mice (Figure S1k,l, Supporting Information) results in increased PPARγ protein level in spinal cord tissue, but not the mRNA (Figure 5c–e). This suggests that ANXA7 does not promote PPARγ gene expression via increasing transcription, but may enhance the stability of PPARγ to avoid its degradation. We then examined the effect of ANXA7 overexpression on PPARγ in vitro. We found that compared to treatment with SEC or ABO alone, combination with ANXA7 overexpression significantly increased PPARγ expression (Figure 5f,g). In addition, the use of GW9662 significantly inhibited the promotion of LDs formation after ANXA7 overexpression (Figure 5h,i). These results indicate that ANXA7 promotes LDs formation and inhibits lipid peroxidation by regulating the intracellular stability of PPARγ. We also examined the regulatory effects of PPARγ on NRF2 in mice with spinal cord injury. The results showed that the expression of NRF2 in mice injected with rosiglitazone was significantly higher than that in mice with spinal cord injury, while suppression of PPARγ activity by GW9662 had the inhibitory effect (Figure 5j,k). In order to demonstrate the importance of PPARγ in ANXA7 regulating process, the combination of ANXA7 overexpression lentivirus and PPARγ inhibitor GW9662 was used. Results showed that inhibition of PPARγ activity counteracts the anti‐oxidative stress effect of ANXA7 with the decreasing expression of PLIN5 and NRF2 (Figure S3, Supporting Information). HE staining indicated that the mice with PPARγ activation had less damage after spinal cord injury (Figure 5l,m). In the results of BMS score measurement and response time to hot stimulation, PPARγ activator also showed a satisfactory effect on neural function recovery (Figure 5n,o).

### 2.6 Activation of PPARγ Promotes LDs Formation and Inhibits Lipid Peroxidation and Oxidative Stress Caused by ANXA7 Knocking Down

**Figure 6:**
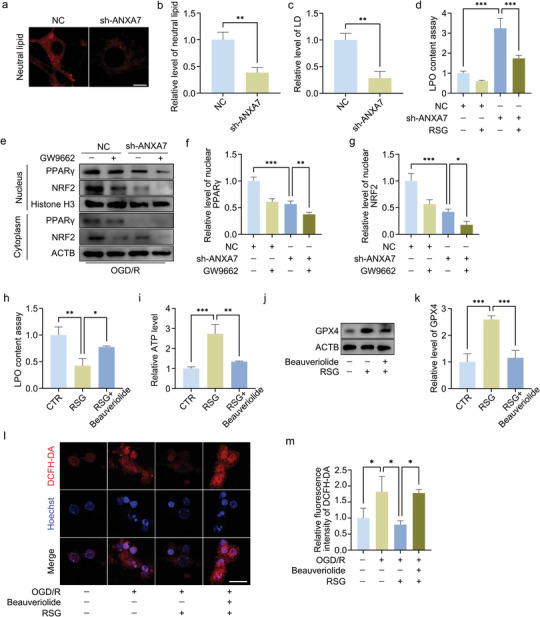
Effect of PPARγ on LDs formation and oxidative stress. a–c) Neutral lipid staining in primary neurons treated with negative control and sh‐ANXA7 lentivirus, and statistical analysis of neutral lipid level and LDs number was performed. Scale bar: 10 µm. d) Primary neurons were treated with negative control or shANXA7 lentivirus, with/without rosiglitazone for 1 h, and lipid peroxidation levels were detected. e–g) The OGD/R model was constructed after primary neurons transfected with negative control or shANXA7 lentivirus were treated with/without GW9662 for 1 h, and nuclear level of PPARγ and NRF2 were detected. h,i) Lipid peroxidation and mitochondrial ATP production were detected by LPO and ATP kits after 1 h of treatment with rosiglitazone and/or Beauveriolide III, respectively. j,k) Western Blot detection of GPX4 protein levels in neurons treated with Rosiglitazone and/or Beauveriolide III for 1 h before OGD/R treatment. l,m) Neurons were treated with rosiglitazone or Beauveriolide III and in combination with rosiglitazone and Beauveriolide III for 1 h, and then OGD/R model was constructed. ROS probe DCFH‐DA was used to detect ROS level. Scale bar: 20 µm. The data were presented as mean ± SD, and Student's T‐test was used for analysis between two groups, while ANOVA was used among more than two groups. ^*^ *p* < 0.05, ^**^ *p* < 0.01, ^***^ *p* < 0.001, n = 3.

We found that neutral lipid levels and LDs formation in neurons were significantly reduced after ANXA7 knocking down. Knocking down ANXA7 also resulted in significantly increased level of lipid peroxidation, which was significantly inhibited by rosiglitazone treatment (**Figure** **6**a–d). We also investigated the nuclear translocation of PPARγ and NRF2 in neurons treated with negative control or sh‐ANXA7 lentivirus respectively. And we found that the nuclear translocation level of PPARγ and NRF2 were significantly inhibited after ANXA7 knocking down, and the nuclear translocation level of NRF2 were further decreased after PPARγ activity was inhibited by GW9662 (Figure 6e–g). This suggests that ANXA7 promotes nuclear translocation of NRF2 and inhibits oxidative stress by regulating PPARγ.

### 2.7 PPARγ Inhibits Lipid Peroxidation and Oxidative Stress by Promoting LDs Formation, and Promotes the Production of ATP in Neurons

Lipid peroxidation leads to mitochondrial permeability conversion disorders and mitochondrial biosynthesis abnormalities, which further lead to energy supply insufficiency, inflammatory environment disorder, oxidative stress level increase, iron death, and induction of apoptosis, etc. Here, Beauveriolide III was used to suppress LDs formation, and we found that inhibition of LDs production could weaken the inhibition of rosiglitazone on lipid peroxidation, and inhibit the increase of ATP level (Figure 6h,i). Moreover, we found that inhibition of LDs formation could inhibit the up‐regulation of GPX4 expression caused by rosiglitazone treatment (Figure 6j,k). ROS probes were used to detect ROS levels in neurons, and the results showed that rosiglitazone could inhibit the increase of ROS levels caused by OGD/R treatment, which was suppressed after inhibition of LDs formation by Beauveriolide III (Figure 6l,m).

### 2.8 ANXA7‐PPARγ Promotes LDs Formation and Interaction with Mitochondria by Regulating PLIN5

**Figure 7:**
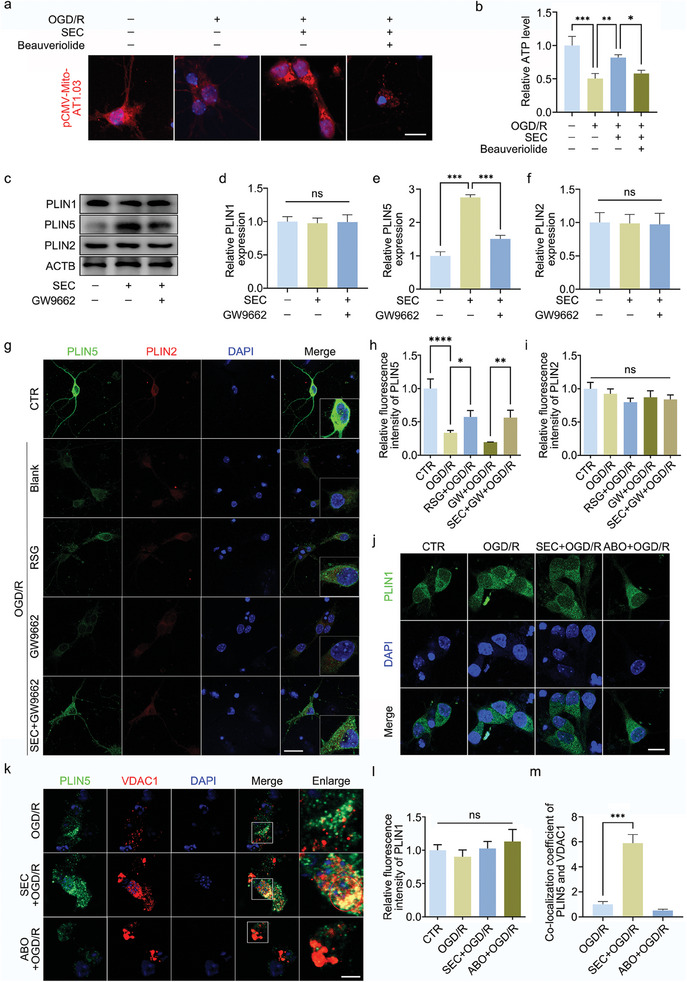
ANXA7‐PPARγ regulates LDs formation by affecting PLIN5 expression. a,b) OGD/R model was constructed after SEC treated neurons for 1 h, and the SEC pretreatment group was treated with Beauveriolide III during reoxygenation processing. Mitochondrial ATP probe pCMV‐Mito‐AT1.03 and ATP test kit were used to detect the level of ATP in neurons. Scale bar:10 µm. c–f) After treatment by SEC with/without GW9662 for 1 h, OGD/R model was constructed, and the levels of PLIN1, PLIN2, and PLIN5 were detected by WB. g) Neurons were treated with rosiglitazone, GW9662, or combination with SEC and GW9662, and OGD/R treatment was conducted. The immunofluorescence was used to detect the expression of PLIN2 and PLIN5. Scale bar: 20 µm. h,i) The quantization analysis of PLIN5 and PLIN2 fluorescence. j) Neurons were treated with SEC or ABO for 1 h, the expression of PLIN1 were detected by immunofluorescence. Scale bar: 10 µm. k) Neurons were treated with SEC or ABO for 1 h, OGD/R model was constructed, and the co‐localization level of PLIN5 and VDAC1 were detected by immunofluorescence. Scale bar: 10 µm. l) The quantization analysis of PLIN1 fluorescence. m) The quantization result of the colocalization coefficient of the above processing. The data were presented as mean ± SD, and ANOVA was used for analysis. ^*^ *p* < 0.05, ^**^ *p* < 0.01, ^***^ *p* < 0.001, n = 3.

Lipid droplet is an important organelle in various cellular activities. In this study, we found that SEC could promote mitochondrial ATP production and inhibit mitochondrial energy deficit caused by OGD/R treatment, while Beauveriolide III could inhibit the effect of SEC and the increase of mitochondrial ATP (**Figure** **7**a,b). Perilipin families were regulated by PPARγ, and members of Perilipin were involved in the process of LDs formation and the interaction between LDs with other organelles. Perilipin5 (PLIN5) is a key protein that mediates the interaction between LDs and mitochondria. Thus, we hypothesized that ANXA7 regulates the expression of the PLIN protein families by regulating PPARγ, which takes part in LDs formation and interaction with mitochondria. As shown in results, SEC promoted the expression of PLIN5, while GW9662 could inhibit the effect (Figure 7c–f), but PLIN1 and PLIN2 protein levels were not significantly affected. Immunofluorescent staining in neurons of PLIN1, PLIN2, and PLIN5 also showed a similar result (Figure 7g–j,l), further proved by the detection of GPX4 and PLIN5 expression in vivo (Figure S4, Supporting Information). To scrutinize whether the interaction between LDs and mitochondrion was mediated by PLIN5, we then used immunofluorescence to detect the co‐localization of PLIN5 and mitochondrial outer membrane protein VDAC1. Results showed that while SEC promotes the co‐localization of PLIN5 with mitochondria, ABO had no significant effect (Figure 7k,m). Therefore, we suggest that ANXA7/PPARγ could regulate LD formation, lipid peroxidation and mitochondrial productivity by promoting the expression of PLIN5.

### 2.9 Regulation of PPARγ Expression Affects Neural Function Recovery after Spinal Cord Injury

**Figure 8:**
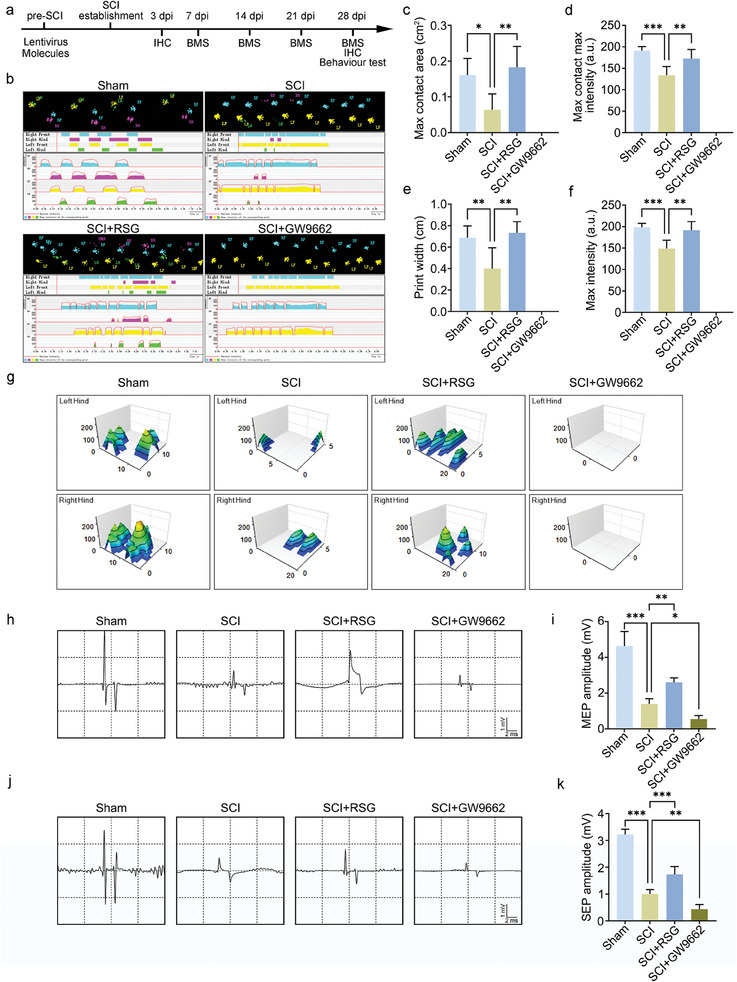
Effect of PPARγ on neural function recovery. a) The flow chart of animal experiments. b) Gait analysis of mice in group treated with sham, SCI, SCI+ rosiglitazone, and SCI+GW9662. c–f) Footprint parameters about max contact area, max contact max intensity, print width, and max intensity from mice treated with sham, SCI, SCI+ rosiglitazone, and SCI+GW9662. g) Area and pressure analysis of footprint from mice treated with sham, SCI, SCI+ rosiglitazone, and SCI+GW9662. h: Motor‐evoked potential (MEP) of mice in group treated with sham, SCI, SCI+ rosiglitazone, and SCI+GW9662. i) Statistical analysis about MEP amplitude. j) Sensory‐evoked potential (SEP) of mice in group treated with sham, SCI, SCI+ rosiglitazone, and SCI+GW9662. k) Statistical analysis about SEP amplitude. The data were presented as mean ± SD, and ANOVA was used for analysis. ^*^ *p* < 0.05, ^**^ *p* < 0.01, ^***^ *p* < 0.001, n = 5.

In the in vitro experiments above, it was demonstrated that ANXA7 could increase stability of PPARγ and thus decreasing neuron injury. But the neuronal protective effect of PPARγ still needs to be explored in vivo. The spinal cord injury model was established and behavior and histological detection were conducted according to the flow chart (**Figure** **8**a). Gait analysis reflects the motion recovery of mice suffered from spinal cord injury. As shown in the results, mice from Sham group had a very regular gait but the SCI group had an irregular gait. The gait of mice treated with SCI and rosiglitazone was more regular than that treated with SCI only, while the group treated with SCI and GW9662 had a worse gait, even no footprint from hindlimbs detected (Figure 8b). Similar results were obtained by measuring gait parameters, such as max contact area, max contact max intensity, print width, and max intensity (Figure 8c–f), as well as analysis about the area and pressure of hindlimbs (Figure 8g). In order to indicate the motor recovery condition further, the electrophysiological detection was conducted. Motor‐evoked potential (MEP) was used to reflect neuromotor function recovery, while sensory‐evoked potential (SEP) was used for neurosensory function recovery. The MEP amplitude decreased significantly after spinal cord injury, and improved after rosiglitazone treatment. However, GW9662 treatment would further suppress the MEP amplitude (Figure 8h,i). The results of SEP detection were similar to MEP (Figure 8j,k), indicating that regulation of PPARγ could affect neural function recovery in vivo.

### 2.10 Regulation of PPARγ Expression Affects Histological Repair of Spinal Cord

**Figure 9:**
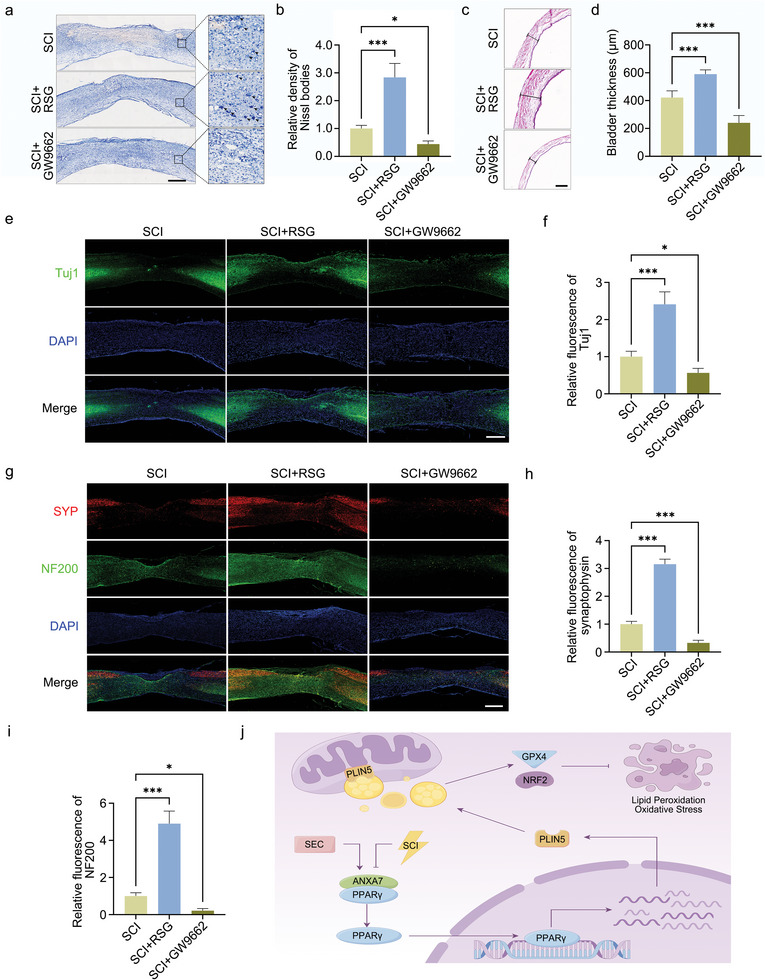
Effect of PPARγ regulation on spinal cord tissue repair. a) Nissl staining of spinal cord tissues 4 days after injury in group SCI, SCI+ rosiglitazone, and SCI+GW9662. Scale bar: 500 µm. b) Statistical analysis about density of Nissl bodies. c) HE staining of bladder from mice in group SCI, SCI+ rosiglitazone, and SCI+GW9662. Scale bar: 250 µm. d) Statistical analysis about bladder thickness. e) Tuj1 fluorescence staining on spinal cord tissues 28 days after injury. Scale bar: 500 µm. f) Statistical analysis about Tuj1 fluorescence intensity. g) Synaptophysin and NF200 fluorescence staining on spinal cord tissues 28 days after injury. Scale bar: 500 µm. h,i) Statistical analysis about synaptophysin and NF200 fluorescence intensity. j) Schematic diagram about regulatory mechanism. The data were presented as mean ± SD, and ANOVA was used for analysis. ^*^ *p* < 0.05, ^***^ *p* < 0.001, n = 5.

To exam the effect of PPARγ on tissue repair, histological staining was conducted. Nissl staining was used to detect neuronal vitality 4 days after injury, and the results indicated that PPARγ activator rosiglitazone has a significant neuroprotective effect and higher density of Nissl bodies, but the inhibitor GW9662 has an opposite effect (**Figure** **9**a,b). Bladder function is an important indicator of neural function recovery, HE staining of bladder tissues was conducted and the thickness was measured. The bladder thickness was increased in mice of rosiglitazone treatment group compared with SCI only, while the thickness was decreased after the inhibitor treatment (Figure 9c,d). The tissue integrality of the spinal cord was detected by Tuj1 immunofluorescence staining and it can be seen that PPARγ activator rosiglitazone could improve the integrality, but the inhibitor had a deleterious effect (Figure 9e,f). Synaptophysin and NF200 fluorescence staining were used to indicate neural junction in the spinal cord after injury. Similar to the results of Tuj1 staining, PPARγ activator rosiglitazone is also beneficial to neural junction after SCI (Figure 9g–i).

## 3 Discussion

The repair of spinal cord injury has always been a difficult problem in the medical field. After the irreversible nerve damage caused by primary spinal cord injury, a series of complex secondary injury reactions will be triggered, including inflammatory immune response, neuronal and glial cell death, and glial scar formation.^[^ ^26^ ^]^ Technical studies on spinal cord injury mostly focus on exosome injection, electrical stimulation, tissue engineering material repair, and stem cell transplantation,^[^ ^27^, ^28^, ^29^ ^]^ while mechanism studies mostly focus on neuronal death, neuroinflammation, and glial scar formation. The absence of related studies about lipid metabolism after spinal cord injury contributes to the insufficient discovery of new targets. Therefore, there is currently a lack of specific treatment with satisfactory effect for its pathological mechanism.

Up to now, studies have shown that neurons of adults cannot regenerate, so it is particularly important to find treatment targets that repair damaged neurons after spinal cord injury specifically. LDs are not just placed where cells store lipids. Increasingly, studies have shown that LDs regulate cellular oxidative stress and signal transduction.^[^ ^30^, ^31^, ^32^, ^33^ ^]^ Previous studies have pointed out that evidence about LDs formation in neurons is limited,^[^ ^34^, ^35^ ^]^ and lipid toxicity after nerve injury is mainly buffered by Schwann cells and microglia. We not only found a large number of LDs in primary cortical neurons from mice cultured for one week but also found that LDs of cortical neurons have vital positive regulation on spinal cord injury. It provides a new idea for the treatment of spinal cord injury.

LDs have been found to play an important role in bone development and a variety of bone‐related diseases. In Ananya Nandy's research, the energy generated by the lipolysis of LDs during osteoblast maturation is critical for bone formation. However, inhibiting the lipolysis of LDs can impair the function of osteoblasts, resulting in impaired bone formation and cortical bone loss.^[^ ^36^ ^]^ Lipid peroxidation leads to mitochondrial permeability conversion disorders and mitochondrial biosynthesis abnormalities, which cause energy supply insufficiency, inflammatory environment disorder, oxidative stress, and induction of ferroptosis apoptosis.^[^ ^37^, ^38^ ^]^ Recently, many studies have mentioned that LDs in different cells, such as microglia, HT22 cell lines, and myeloid cells, have essential regulatory effect on spinal cord injury and central nervous system.^[^ ^39^, ^40^, ^41^, ^42^, ^43^ ^]^ And yet, we focus more on the role and function of LDs in primary neurons. We further found that inhibition of LDs production could weaken the inhibition of rosiglitazone on lipid peroxidation and inhibit the increase of ATP level and ROS clearance in cells induced by rosiglitazone. LDs and mitochondria are functionally close related. And LDs can promote its utilization through interaction with mitochondria, further promoting mitochondrial productivity and inhibiting mitochondrial damage.^[^ ^44^ ^]^ The perilipin families are important protein components of LDs and they are regulated by PPARγ transcription. They take part in the process of formation and interaction with other organelles of LDs.^[^ ^45^, ^46^ ^]^ Perilipin5 (PLIN5) is a key protein that mediates the interaction between LDs and mitochondria.^[^ ^47^, ^48^ ^]^

Our previous studies have shown that activation of ANXA7 inhibits neuronal apoptosis by regulating autophagy, thereby repairing SCI. However, we are still investigating the comprehensive mechanism of ANXA7 in regulating SCI. We found for the first time that ANXA7 can co‐regulate neuronal lipid peroxidation through interaction with PPARγ, and activation of ANXA7 could inhibit oxidative stress and lipid peroxidation by promoting expression of PPARγ, thus repairing spinal cord injury (Figure 9j). In addition, we found that LDs in primary neurons are involved in the regulation of spinal cord injury. ANXA7‐PPARγ signaling axis can repair mitochondrial dysfunction by regulating LDs formation and interaction with mitochondria to buffer lipid peroxidation. Furthermore, ANXA7 also promoted the nuclear translocation of NRF2 and the expression of GPX4 to inhibit oxidative stress and lipid toxicity. Combined with our published results, this suggests that ANXA7 promotes SCI repair by regulating different pathways to repair injured nerves. ANXA7 activator has a promising application in the treatment of SCI.

## 4 Conclusion

In summary, we discovered that ANXA7 could interact with PPARγ to promote its stability. SEC, the activator of ANXA7, promotes nuclear translocation of PPARγ, thus promotes LDs formation and regulates the interaction with mitochondria by promoting expression of PLIN5, and inhibits oxidative stress and lipid peroxidation. Moreover, ANXA7/ PPARγ could also inhibit oxidative stress and lipid peroxidation by promoting nuclear translocation of NRF2 and expression of GPX4. Hence, our study proposes a new mechanism for the regulation of LDs metabolism by ANXA7/ PPARγ, and also puts up a new specific target for SCI repair.

## 5 Experimental Section

### Cell Culture and Treatments

C57BL/6J pregnant mice (E17‐19 days) used for primary neurons extraction. After removing the vascular membrane on the brain, the cerebral cortex was dissected and washed with high‐glucose DMEM (BasalMedia, Cat# L110KJ) on the ice. The cerebral cortex tissues were cut into ≈1 mm^3^ pieces and collected, then they were digested at 37 °C with papain (2 mg mL^−1^, Worthington, Cat# LS003126) containing DNase. To get single cells, 40 µm strainer was used. The plates or dishes were precoated with poly‐l‐lysine, and neurons were seed with high‐glucose DMEM containing 10% fetal bovine serum (CellMax, Cat# SA101.02) and 1% PS. About 4 h later, DMEM medium was discarded and replaced with neurobasal medium with 2 mmol L^−1^ glutamine, 1% B27, and 1% PS.

### Oxygen–Glucose Deprivation/Reoxygenation Treatment

The neurobasal medium was discarded and the neurons were washed for three times. Glucose‐free DMEM (BasalMedia, L160KJ) was used for the construction of oxygen–glucose deprivation/reoxygenation model. Cells were placed in a 37 °C incubator with 1% O_2_, 94% N_2_, and 5% CO_2_ for 1 h to induce injury. The medium was then replaced with normal neurobasal medium and cultured in normal incubatory conditions for 3 h. Cells were treated with small compounds 1 h before OGD/R treatment, with the concentration of 20 µm SEC, 10 µm ABO, 20 µm rosiglitazone, 3 µm GW9662 and 10 µm Beauveriolide III.

### CCK‐8 Assay

In order to investigate the survival ratio of neurons, cell counting kit‐8 (Beyotime, C0038) was used according to the manufacturer's instructions. OD450 was measured to reflect the viability of neurons and each experiment was measured three times.

### Western Blotting

Cells were lysed with RIPA buffer containing protease and phosphatase inhibitor cocktail and performed ultrasonication for further lysis. Then the protein suspension was centrifuged at 12 000 rpm for 25 min. The supernatant was collected and boiled for 5 min after adding the 5× loading buffer. SDS‐PAGE electrophoresis was used for proteins separation, after which, the protein was electrotransferred onto the PVDF membrane. The membrane was blocked with 5% BSA and incubated with following primary antibodies 4 °C overnight: Annexin A7 (Proteintech, Cat# 10154‐2‐AP), PPARγ (Abcam, Cat# ab178860), GPX4 (Cell signaling technology, Cat# #52 455), NRF2 (Cell signaling technology, Cat# 12721S), PLIN1 (Abcam Cat# ab172907), PLIN2 (Santa Cruz, Cat# sc‐377429), β‐actin (CST, Cat# 4970S), PLIN5 (Proteintech, Cat# 26951‐1‐AP), Histone H3 (Proteintech, 17168‐1‐AP). Then, the membrane was washed with TBST for three times and incubated with secondary antibodies at 37 °C for 1 h. Protein bands were visualized by using electrochemiluminescence reagent. For nuclear protein detection, the nuclear protein and cytoplasmic protein extraction kit (Thermo Fisher, Cat# 78 833) was used.

### Co‐Immunoprecipitation

Cells were lysed with lysis buffers and the BCA kit was used to quantify the protein concentration. The supernatant with total protein content >500 µg was incubated with specific antibodies or normal IgG at 4 °C overnight, followed by the addition of pre‐cleared protein A/G agarose beads and incubation at 4 °C for 3 h. Then, the beads were collected and washed three times. The samples were boiled after adding 2× SDS loading buffer to release proteins. The input and the immunoprecipitated fraction were analyzed by western blotting.

### RNA Isolation and Quantification

Total RNA was collected by using the Total RNA Extraction Kit (Solarbio Life Sciences). The cDNA was reverse transcripted with Revert Aid First Strand cDNA Synthesis Kit (Thermo Fisher), and amplificated with Pro Taq HS SYBR Green (Accurate Biotechnology (Hunan) Co.,Ltd, ChangSha, China, Cat# AG11701) to reflect mRNA content. And endogenous β‐actin was used to normalization. Sequences of primer were listed as follows: *Actin* forward 5′‐ACCTCTTTTGGATTGGGCTTCA‐3′, reverse 5′‐ ATGGACGGTGAAGAAGTTGC‐3′; *Nrf2* forward 5′‐ TCTTGGAGTAAGTCGAGAAGTGT‐3′, reverse 5′‐GTTGAAACTGAGCGAAAAAGGC‐3′; *Ho‐1* forward 5′‐ AAGCCGAGAATGCTGAGTTCA‐3′, reverse 5′‐ GCCGTGTAGATATGGTACAAGGA‐3′; *Gpx4* forward 5′‐ CCCGATATGCTGAGTGTGGTTTAC‐3′, reverse 5′‐ TTTCTTGATTACTTCCTGGCTCCTG‐3′; *Pparγ* forward 5′‐TCGCTGATGCACTGCCTATG‐3′, reverse 5′‐GAGAGGTCCACAGAGCTGATT‐3′.

### LPO Content Assay

Cells were collected and the supernatant was discarded. After adding the extraction liquid (it is recommended that 5 million cells with 1 mL extraction solution), the cells were lysed by ultrasound, and the supernatant was collected after centrifugation. The supernatant was measured by LPO Assay Kit (Sangon Biotech, D799602‐0050) followed with instructions.

### MDA Detection

Cells were lysed and centrifugated with 12000 g at 4 °C for 10 min and the protein concentration of supernatant was determined by BCA assay kit. The supernatant was measured with MDA detection kit (Beyotime, S0131S) followed with instructions.

### Prediction of Proteins Interaction

The FASTA sequences of ANXA7 and PPARγ proteins were obtained from NCBI and changed to PDB format through Swiss‐Model website. After loading the sequence information to Vasker Lab, the interaction between above two proteins was predicted and a PDB file containing the interaction information was created. PBDePISA website could help to realize visualization.

### Spinal Cord Contusion Injury Model Establishment

The study was approved by the Ethics Committee in Qilu Hospital of Shandong University. The procedure of spinal cord contusion injury model establishment was described in our previous publication.^[^ ^18^ ^]^ Briefly, 8 weeks old female C57BL/6J mice (n = 5 per group) were used. The mice were anaesthetized with isoflurane. T10 vertebral level was exposed, and the mice received a moderate (75 kdyne) contusion SCI using the Infinite Horizons impactor (Precision Systems and Instrumentation, LLC.). The bladder was emptied manually at least twice daily after SCI model establishment. 3 days before spinal cord injury model establishment, lentivirus (bought from Beijing SyngenTech Co., Ltd.) was injected in situ into spinal cord. Three points were taken from each side of the mouse T10 spinal cord segment, and 1×10^6^ TU lentivirus were injected into each point using a microinjector. For small molecules administration, intraperitoneal injection was conducted with concentration of rosiglitazone 5 mg kg^−1^, GW9662 1 mg kg^−1^ and SEC 10mg kg^−1^.

### Catwalk Gait Analysis

Gait analysis was performed 4 weeks after SCI. Mice in each group were evaluated by the Catwalk‐assisted gait analysis (Noldus Inc., Wageningen, Netherlands). When the mice crossed the pathway, their paws prints were recorded by CatWalkTM XT 10.6 software automatically. Each mouse performed three runs to ensure stability. The parameters and figures were exported from the software and used for statistics.

### Electrophysiological Analysis

After anesthesia, the skull of mice was drilled, and the brain was exposed. The stimulation electrode was placed on the brain, the receiving electrode was placed in the opposite sciatic nerve, and the grounding electrode was clamped into the skin. The motor‐evoked potential (MEP) was analyzed by recording amplitude with electrophysiological device (Zhuhai Yiruikeji Co, Ltd.). Positions of the stimulation and receiving electrode were exchanged and the sensory‐evoked potential was recorded.

### Locomotor Function Investigation

The motor functions of hind limbs in mice were evaluated 0, 1, 7, 14, 21, and 28 days after surgery. The mice were allowed to walk freely in an open field, and BMS score was evaluated by blinded observations.

### Immunofluorescence

Cells were seed on the glass‐bottom dishes (NEST Biotechnology Co. Ltd., Wuxi, China) and fixed with 4% paraformaldehyde and permeabilized with 0.1% Triton X‐100 after treatment. After blocking with 5% BSA, the samples were incubated with primary antibodies at 4 °C overnight: NRF2 (Cell signaling technology, Cat# 12721S), HO‐1 (Abcam, Cat# ab189491), PPARγ (Abcam, Cat# ab178860), ANXA7 (Proteintech, Cat# 10154‐2‐AP), PLIN5 (Proteintech, Cat# 26951‐1‐AP), PLIN2 (Santa Cruz, Cat# sc‐377429), PLIN1(Abcam, Cat# ab172907), VDAC1 (Proteintech, Cat# 66345‐1‐Ig). After washing with TBST, the samples were incubated with secondary antibodies at 37 °C for 1 h and DAPI was used for showing nucleus. The fluorescence signals were detected by Zeiss LSM900 confocal microscope.

### Immunohistochemistry

The mice were perfused with 4% paraformaldehyde under anesthesia, spinal cord tissues were dissected and fixed in 4% paraformaldehyde solution and dehydrated in sucrose solution. Then the tissues were embedded with TissueTek OCT compound and sliced into 10 µm pieces. The samples were incubated with primary antibodies after permeabilizing and blocking with 5% BSA containing 0.1% Triton X‐100: NeuN (Abcam, Cat# ab177487), Annexin A7 (Proteintech, Cat# 10154‐2‐AP), NF200(Proteintech, Cat#18934‐1‐AP), synaptophysin (Abcam, Cat# ab32127). Corresponding fluorescent secondary antibodies were incubated at room temperature for 1 h, and images were acquired through LSM900 confocal microscope after nucleus staining with DAPI.

### Histological Analysis

Hematoxylin and eosin (H&E) and Nissl staining were carried out for histological analysis. The sections were washed in PBS. H&E and toluidine blue kits were used for the H&E and Nissl staining respectively according to instruction. The images were captured and analyzed.

### Statistical Analysis

All the experiments were repeated at least three times. The data was presented as mean ± SD. The distribution of each dataset was examined using the Shapiro–Wilk test to confirm normality before statistical analysis. The differences between two groups were evaluated by Student's T‐test, and one‐way analysis of variance (one‐way ANOVA) with Tukey's post hoc test was used for analyzing differences among more than two groups. The *P* value <0.05 was regarded as significant differences. Statistical software SPSS 20.0 was used for statistical analysis.

## Conflict of Interest

The authors declare no conflict of interest.

## Author Contributions

L.C. and H.L. contributed equally to this work. L.C., N.L., and S.F. contributed to the project conception and experiments design. The experiments performance and data analyzation were mainly performed by L.C. and H.L. L.J., Z.W., and Y.C. provided analysis and interpretation of data. N.L. and S.F. won the financial support. The manuscript was written by N.L. and L.C., and other authors conducted the review. All authors have read and agreed to the published version of the manuscript.

## Supporting information



Supporting Information

## Data Availability

The data that support the findings of this study are available from the corresponding author upon reasonable request.
