# Supplementary material for: Lipid Droplets Metabolism Mediated by ANXA7‐PPARγ Signaling Axis Regulates Spinal Cord Injury Repair in Mice
Source: Adv Sci (Weinh). 2025 Feb 25;12(16):2417326. doi: 10.1002/advs.202417326 (PMC12021101; doi:10.1002/advs.202417326)
Supplement: Supplementary file 1 — Supporting Information [file ADVS-12-2417326-s001.pdf]

ADVANCED SCIENCE
Open Access

Supporting Information

for *Adv. Sci.*, DOI 10.1002/advs.202417326

# Lipid Droplets Metabolism Mediated by ANXA7-PPAR$\gamma$ Signaling Axis Regulates Spinal Cord Injury Repair in Mice

*Lu Chen, Haoran Liu, Linlin Jiang, Zihang Wang, Yong Chang, Na Li* and Shiqing Feng**

Supplementary information to

# Lipid Droplets Metabolism Mediated by ANXA7-PPARγ Signaling Axis Regulates Spinal Cord Injury Repair in Mice

Lu Chen[1,*], Haoran Liu[2,*], Linlin Jiang[1], Zihang Wang[2], Yong Chang[1], Na Li[1] and Shiqing Feng[1,3]

[1]Orthopaedic Research Center of Shandong University, Department of orthopaedics, Qilu Hospital of Shandong University, #107 Wenhua West Road 250012, Jinan, Shandong Province, China

[2]School of Basic Medical Sciences, Shandong University, #44 Wenhua West Road 250012, Jinan, Shandong Province, China

[3]The Second Hospital of Shandong University, Jinan, Shandong, Province, China

Corresponding Author:

Na Li, Email: lina09612@qiluhospital.com

Shiqing Feng, Email: shiqingfeng@sdu.edu.cn

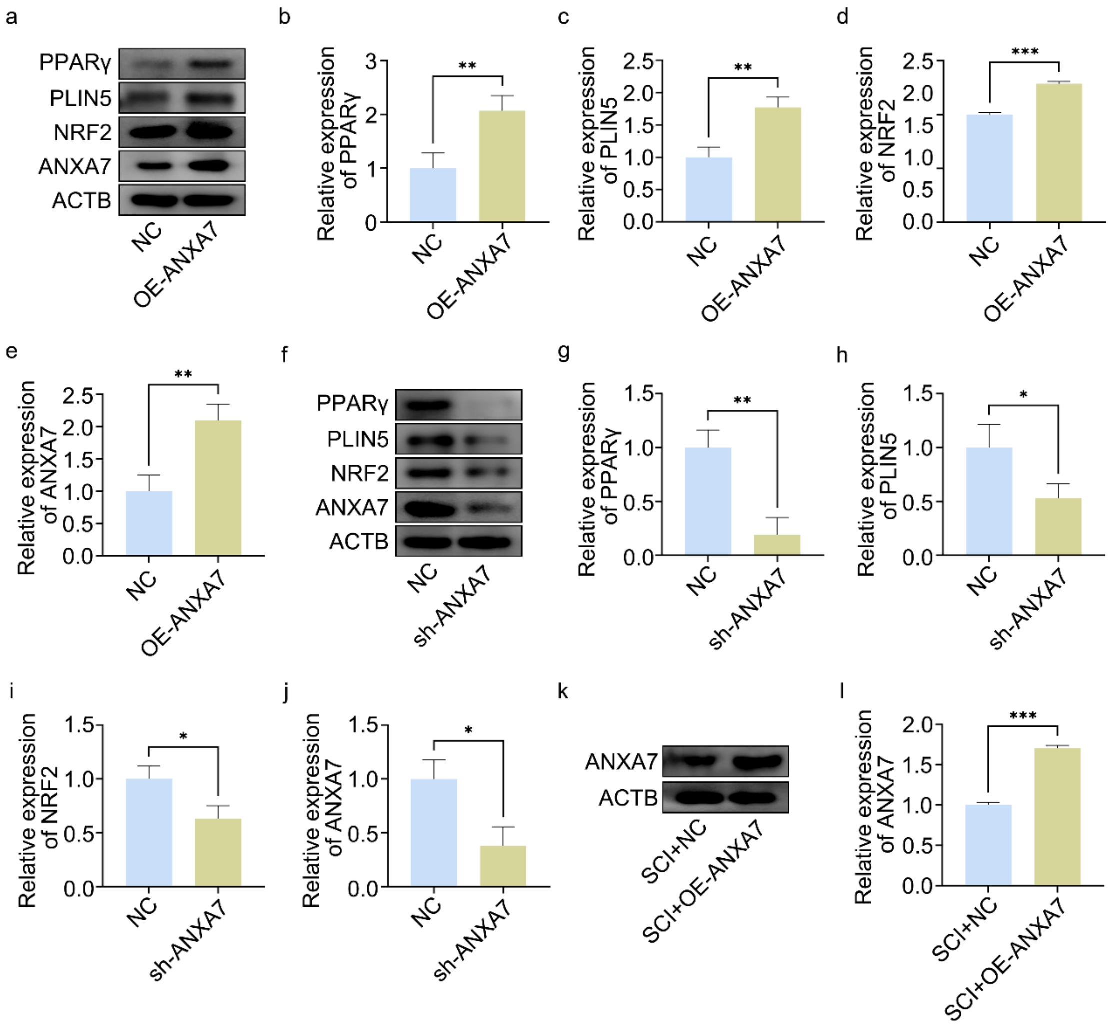


Figure S1. ANXA7 could affect oxidative stress related protein expression. a-e: The protein levels of PPARγ, PLIN5, NRF2 and ANXA7 were detected by WB after OGD/R in primary neurons treated with negative control and ANXA7 overexpression lentivirus. f-j: The protein levels of PPARγ, PLIN5, NRF2 and ANXA7 were detected by WB after OGD/R in primary neurons treated with negative control and ANXA7 interference lentivirus. k-l: The protein levels of ANXA7 in spinal cord treated with negative control and ANXA7 overexpression lentivirus. The data were presented as mean ± SD, and Student's T-test was used for analysis between two groups. *$P$ < 0.05, **$P$ < 0.01, ***$P$ < 0.001, n = 3.

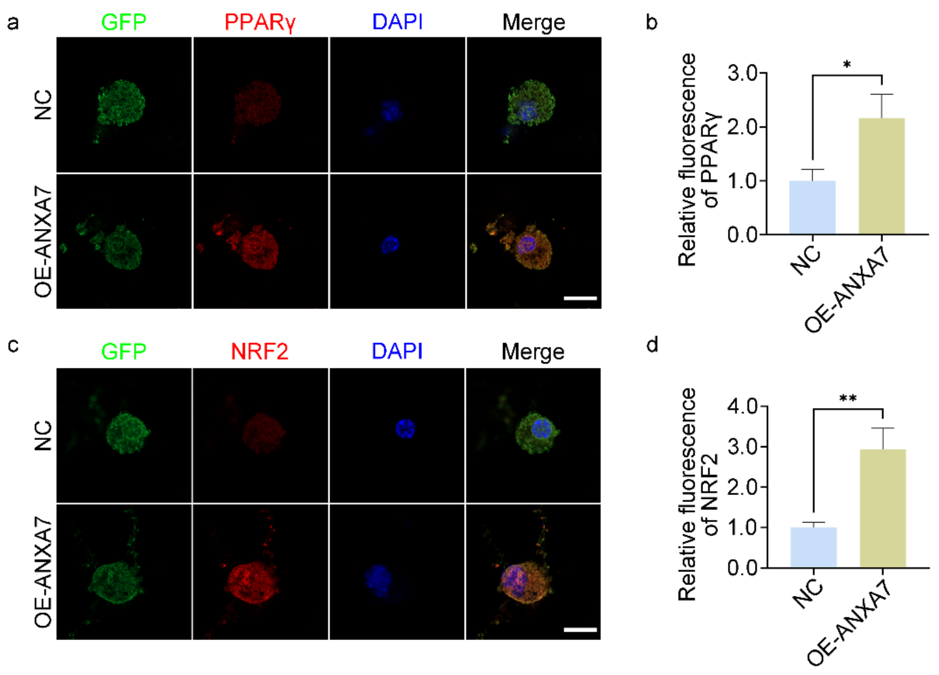


Figure S2. Overexpression of ANXA7 could increase the expression of PPARγ and NRF2. a-b: The expression level of PPARγ was detected after OGD/R in primary neurons treated with negative control or ANXA7 overexpression lentivirus. Scale bar: 10 μm. c-d: The expression level of NRF2 was detected after OGD/R in neurons treated with negative control or ANXA7 overexpression lentivirus. Scale bar: 10 μm. The data were presented as mean ± SD, and Student's T-test was used for analysis between two groups. $*P < 0.05$, $**P < 0.01$, n = 3.

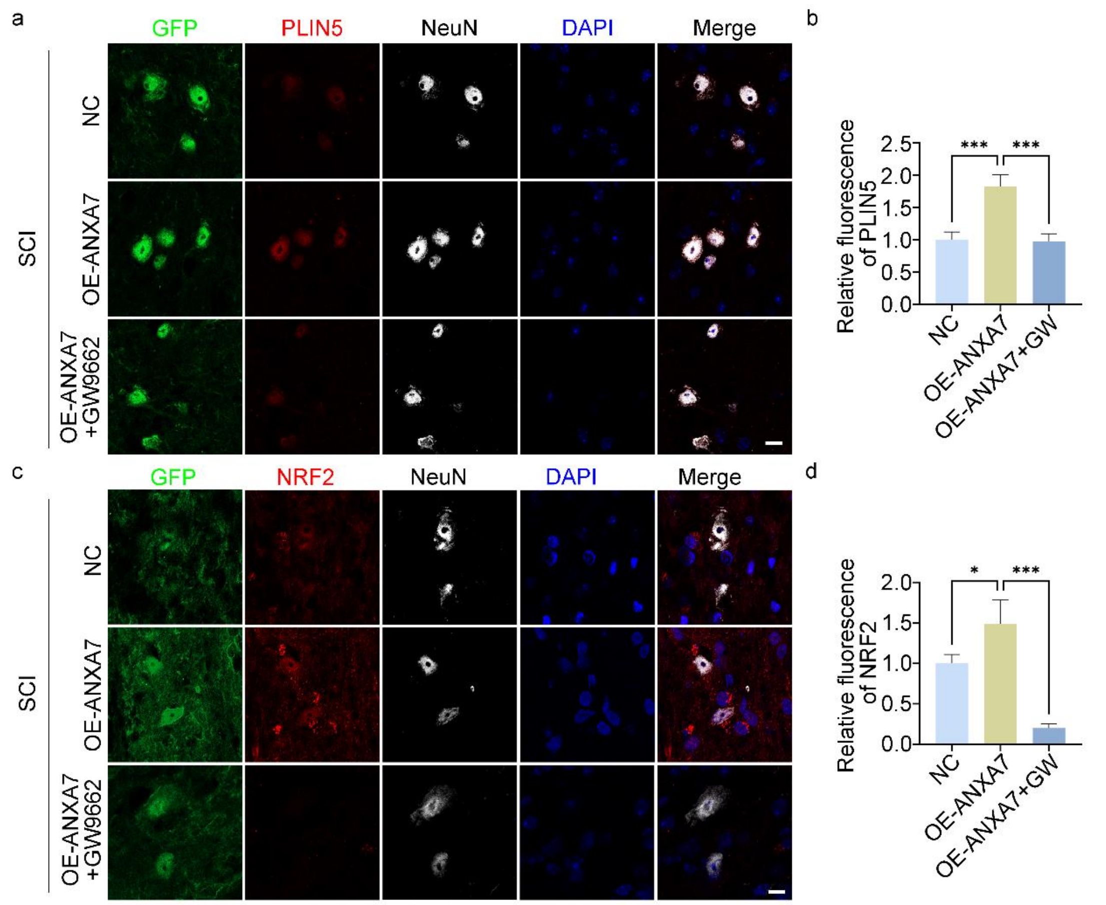


Figure S3. Inhibition of PPARγ activity counteracted the effect of ANXA7. a-b: The expression level of PLIN5 in neurons was detected by IHC 4 days after SCI in groups treated with negative control, ANXA7 overexpression lentivirus or combination with ANXA7 overexpression lentivirus and GW9662. Scale bar: 10 μm. c-d: The expression level of NRF2 in neurons was detected by IHC 4 days after SCI in groups treated with negative control, ANXA7 overexpression lentivirus or combination with ANXA7 overexpression lentivirus and GW9662. Scale bar: 10 μm. The data were presented as mean ± SD, and ANOVA was used for analysis. *$P$ < 0.05, ***$P$ < 0.001, n = 3.

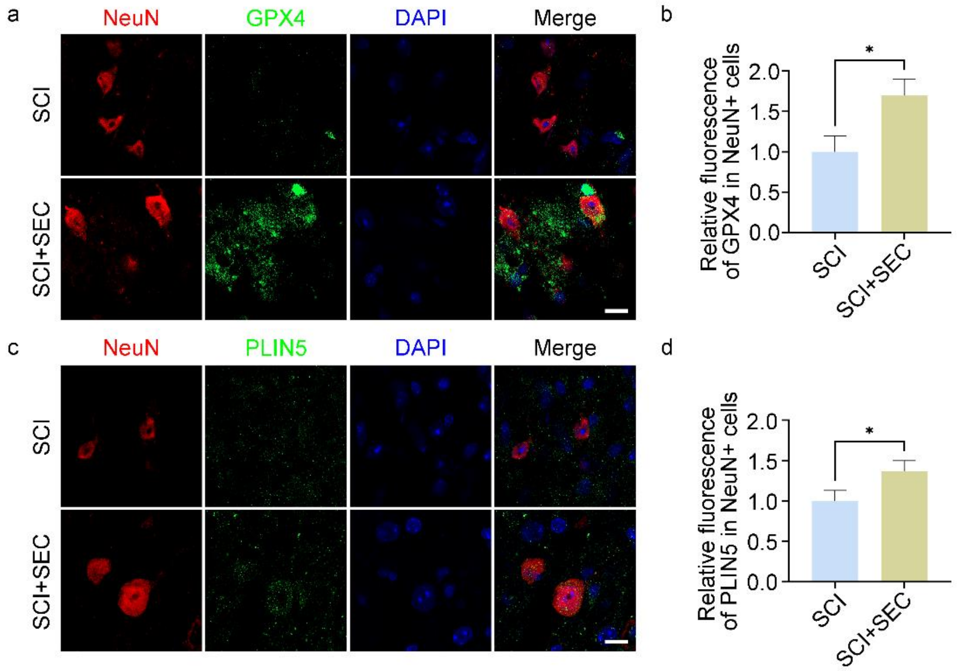


Figure S4. The effect of ANXA7 activator SEC on GPX4 and PLIN5 in neurons after SCI. a-b: The expression level of GPX4 in neurons was detected by IHC 4 days after SCI in groups treated with or without SEC. Scale bar: 10 µm. c-d: The expression level of PLIN5 in neurons was detected by IHC 4 days after SCI in groups treated with or without SEC. Scale bar: 10 µm. The data were presented as mean ± SD, and Student's T-test was used for analysis between two groups. *$P < 0.05$, n = 3.
